# Multiply periodic states and isolated skyrmions in an anisotropic frustrated magnet

**DOI:** 10.1038/ncomms9275

**Published:** 2015-09-23

**Authors:** A. O. Leonov, M. Mostovoy

**Affiliations:** 1 Zernike Institute for Advanced Materials, University of Groningen, Nijenborgh 4,9747 AG Groningen, The Netherlands

## Abstract

Multiply periodic states appear in a wide variety of physical contexts, such as the Rayleigh–Bénard convection, Faraday waves, liquid crystals and skyrmion crystals recently observed in chiral magnets. Here we study the phase diagram of an anisotropic frustrated magnet which contains five different multiply periodic states including the skyrmion crystal. We clarify the mechanism for stabilization of these states and discuss how they can be observed in magnetic resonance and electric polarization measurements. We also find stable isolated skyrmions with topological charge 1 and 2. Their spin structure, interactions and dynamics are more complex than those in chiral magnets. In particular, magnetic resonance in the skyrmion crystal should be accompanied by oscillations of the electric polarization with a frequency depending on the amplitude of the a.c. magnetic field. These results show that skyrmion materials with rich physical properties can be found among frustrated magnets. We formulate rules to help the search.

## Abstract (web summary)

Skyrmions—magnetic vortices with an additional twist—have only been observed in a small number of chiral magnets, all with specific non-centrosymmetric structure. Here, the authors suggest that skyrmions can be found in many frustrated magnets as long as they meet a specific set of criteria.

## Graphical abstract

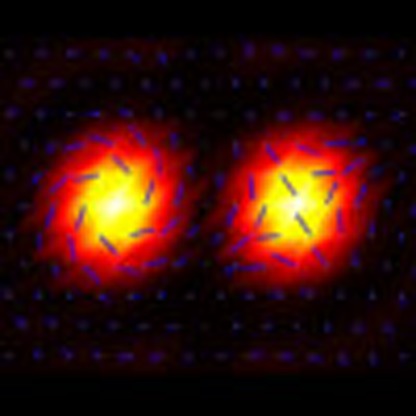

The magnetic skyrmion is a topological defect with a complex non-coplanar spin structure^1^. Predicted over 20 years ago^2^, skyrmions have been recently observed in conducting and insulating helimagnets under an applied magnetic field^3^,^4^. Magnetic skyrmion provides a physical realization of the idea that quantization of physical observables, such as electric and baryon charge, is rooted in topology^5^,^6^. The skyrmion spin texture induces one flux quantum of an effective magnetic field acting on spin-polarized electrons and magnons, which gives rise to topological Hall effects in charge and heat transport, and at the same time sets skyrmions into motion^7^,^8^,^9^,^10^,^11^,^12^. Low critical currents needed to manipulate skyrmions opened a new active field of research—skyrmionics, which has a goal of developing skyrmion-based magnetic memory and data processing devices^13^,^14^,^15^,^16^.

So far, skyrmions have only been found in a handful of materials, all with the non-centrosymmetric cubic B20 structure, in which the relativistic Dzyaloshinskii–Moriya interaction turns a collinear spin state into a helical spiral. Under an applied magnetic field, the spiral transforms into the skyrmion crystal (SkX) with a triangular magnetic superlattice. Further increase of the magnetic field results in a transition to the saturated ferromagnetic (FM) state, in which isolated skyrmions exist as stable topological defects^17^,^18^.

Skyrmions are close relatives of magnetic bubbles (cylindrical domains) and the transformation of the helical spiral into SkX is analogous to the field-induced transition between the stripe domain state and the bubble array taking place in thin FM films. In the framework of Landau theory, the bubble array is described by three coexisting spin modulations with the wave vectors, **q**_1_, **q**_2_ and **q**_3_, which add to zero. This 3*q*-state is stabilized by an anharmonic interaction between these modulations and the uniform magnetization induced by an applied magnetic field^19^. In bulk chiral magnets, SkX competes with the conical spiral (CS) state, and an additional order-from-disorder mechanism—stabilization by thermal spin fluctuations—was invoked to explain why skyrmions are only observed at elevated temperatures^3^,^20^.

Practical applications of skyrmions crucially depend on finding new classes of skyrmion materials and new microscopic mechanisms for their stabilization. Recently, Okubo *et al*.^21^ studied numerically an isotropic Heisenberg spin model on a (centrosymmetric) triangular lattice with competing spin interactions. It shows a spiral ground state, which above a critical magnetic field transforms into the SkX. In addition, a state with two coexisting spirals was found in applied magnetic fields. These multi-*q* states are induced by thermal fluctuations and appear at non-zero temperatures.

Here we discuss a mechanism that lends stability to multiply periodic states even at zero temperature. We show that a uniaxial magnetic anisotropy strongly affects spin ordering in the frustrated triangular magnet: tuning magnetic field and anisotropy at zero temperature, we find eight different phases, five of which are multi-*q* states. A large part of the phase diagram is occupied by the SkX. We clarify the nature of the phases found in numerical simulations and present results of analytical studies of instability of spiral states towards additional periodic modulations. We calculate magnetic resonance spectra and magnetically induced electric polarization, which show strong sensitivity to spin ordering and can be used for experimental identification of complex magnetic states. We explore rich physics of isolated skyrmions in frustrated magnets. Their monopole and toroidal moment densities oscillate with the distance from the skyrmion centre, which gives rise to alternation of attraction and repulsion between skyrmions, clustering of skyrmions in high magnetic fields and stability of skyrmions with topological charge 2. Dynamics of skyrmions in frustrated magnets is different from that of chiral magnets because of the additional collective degree of freedom—skyrmion helicity. The coupling between the helicity and the skyrmion centre-of-mass motion leads to a dynamical magnetoelectric effect.

## Results

### The model

We consider classical spins, **S**_*i*_, of unit length on a triangular lattice in the *xy*-plane with FM nearest-neighbour (NN) and anti-FM next-NN (NNN) exchange interactions:


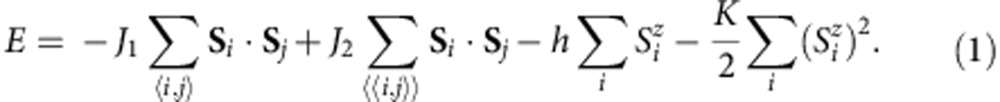


where 〈*i*,*j*〉 and 〈〈*i*,*j*〉〉 denote pairs of NN and NNN spins, respectively, and *J*_1_,*J*_2_>0. The third and the fourth terms describe, respectively, the interaction with the magnetic field parallel to the *z* axis and the single-ion magnetic anisotropy.

The critical ratio *J*_2_/*J*_1_ for appearance of magnetic spirals and other modulated spin states is 1/3. This can be understood by considering a modulation with the wave vector along the *y* axis parallel to a NNN bond (see inset in Fig. 2b), in which case the model effectively reduces to a spin chain running in the *y* direction with the FM NN interaction, 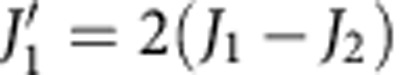, and the anti-FM NNN interaction, 
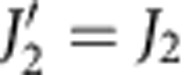
. A spiral state in a chain appears for 
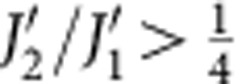
, which corresponds to 
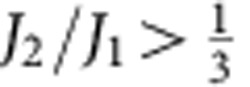
. In what follows, the value of 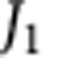 is set to 1.

### Phase diagram

**Figure 1:**
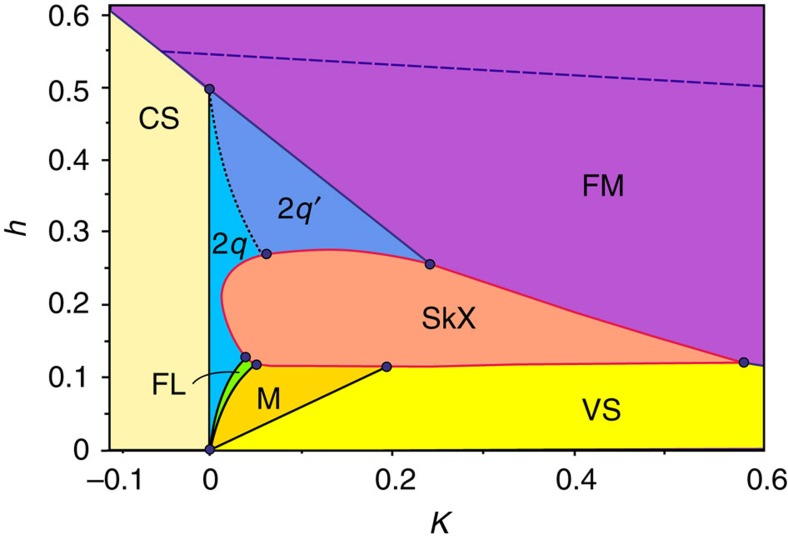
Zero-temperature phase diagram of the frustrated triangular antiferromagnet. The eight phases are as follows: fully polarized ferromagnetic state (FM), conical spiral (CS), 2*q*-state, 2*q′*-state, vertical spiral (VS), VS plus two in-plane sinusoidal modulations (M), flop state (FL) and skyrmion crystal (SkX). Dashed line is the upper critical field, above which isolated skyrmions are unstable. The NNN exchange constant, *J*_2_=0.5, magnetic anisotropy, *K*, and magnetic field, *h*, are measured in units of the NN exchange constant, *J*_1_=1.

**Figure 2:**
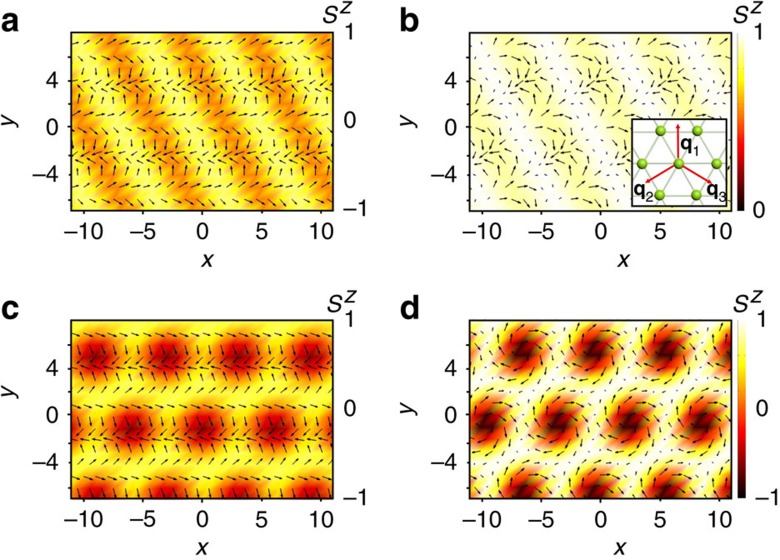
Multi-*q* states. In-plane components (arrows) and out-of-plane components (colour) of spins in four selected states from the phase diagram. (**a**) 2q-state, (**b**) 2*q′*-state, (**c**) flop state and (**d**) skyrmion crystal. The inset shows the wave vectors of the three fundamental modulations in the multi-*q* states.

Figure 1 shows the zero-temperature phase diagram of the model in the (*K*,*h*)-plane for *J*_2_=0.5, which counts eight phases. Their spin configurations can be described in terms of the Fourier series,


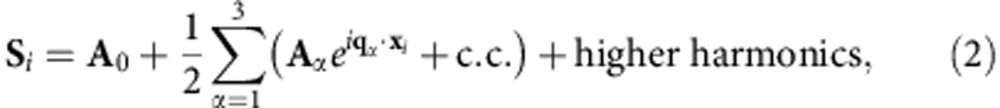


where **q**_1_=*q*(0,1), 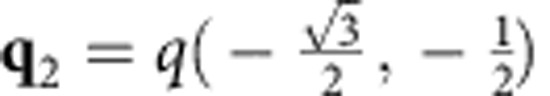 and 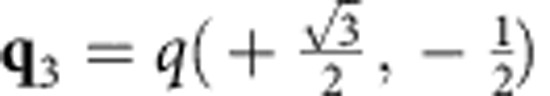 are the wave vectors of the three fundamental modulations minimizing the exchange energy (inset in Fig. 2b), such that **q**_1_+**q**_2_+**q**_3_=0. In all states, except the flop state (FL), the uniform spin component **A**_0_ is parallel to the applied magnetic field: 
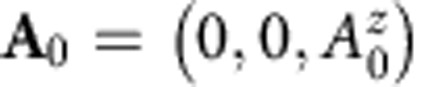
. The amplitudes of higher harmonics, that is, modulations with other wave vectors from the triangular lattice in the reciprocal space spanned on **q**_1_ and **q**_2_, are relatively small. The eight ground states of the model are:

(1) The fully polarized FM state with 
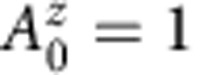
 and **A**_*α*_=0.

(2) The CS state, which occupies the region of *K*<0 (easy plane anisotropy) and 
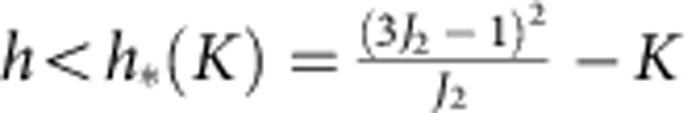
. This is a single-*q* state with only one non-zero **A**_*α*_ describing a circular spiral in the horizontal plane, for example, 
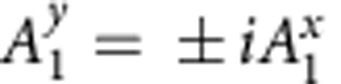
 and 
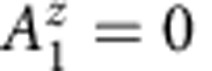
,±sign corresponding to two opposite directions of spin rotation in the spiral.

(3) The 2*q*-state with |**A**_1_|>|**A**_2_|≫|**A**_3_| (Fig. 2a). For *K*>0 (easy axis anisotropy), the conical state acquires the second spiral modulation in the horizontal plane, for example, with the wave vector **q**_2_ and the opposite sense of spin rotation compared with that in the first spiral: 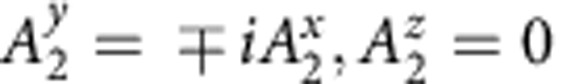. This 2*q*-state has also a small sinusoidal spin modulation parallel to the *z* axis with the wave vector **q**_3_: 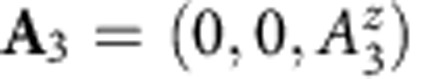. As explained in the next section, the sinusoidal modulation stabilizes the 2*q*-state state. For small positive *K*, 
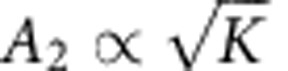
, that is, *K*=0 is a critical line.

(4) The 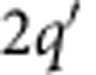-state, which is the same as the 2*q*-state but with |**A**_1_|=|**A**_2_| (Fig. 2b). As *K* increases, the amplitude of the second spiral grows until it becomes equal to that of the first spiral. This transition is marked by dotted line in Fig. 1.

(5) The spiral in a vertical plane, for example, 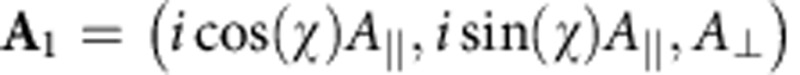 and 
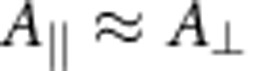
, where the angle-*χ* describes the rotation of the spiral plane around the *z* axis. This is the ground state for low applied fields and *K*>0. The vertical spiral (VS) is not perfectly circular and has higher harmonics.

(6) The spiral in a vertical plane with two sinusoidal modulations in the direction normal to the spiral plane, for example, the spiral in the *yz*-plane with the wave vector **q**_1_ and **A**_1_≈*A*_1_(0,*i*,1) and the sinusoidal modulations with the wave vectors **q**_2_ and **q**_3_ along the *x* direction: **A**_2_=(*A*_2_,0,0) and 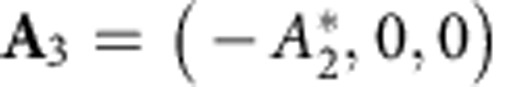. The sinusoidal modulations appear above a critical magnetic field, *h*_1_(*K*), and the transition between the VS state and this mixed (M) is of second order.

(7) The FL (Fig. 2c). As magnetic field increases further, the spiral plane changes from vertical to horizontal. This flop transition is not instantaneous, but occurs in a narrow field interval, *h*_2_(*K*)<*h*<*h*_3_(*K*), in which the spiral plane as well as the direction of the sinusoidal modulations rotate. The orientation of the spiral plane changes abruptly at *h*_2_, and at *h*_3_(*K*) the system undergoes a first-order transition to the 2*q*-state. The FL is the only state, in which the uniform magnetization **A**_0_ has a non-zero component parallel to the *xy*-plane.

(8) The triangular skyrmion (or antiskyrmion) crystal state (Fig. 2d) with equal amplitudes of the three fundamental modulations:


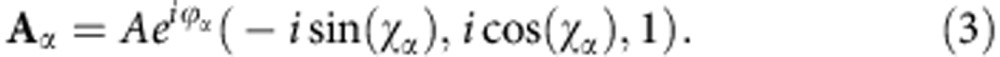


These modulations are spirals in three vertical planes rotated with respect to each other by ±120°: 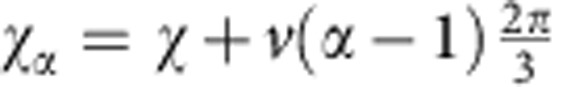 (*α* = 1,2,3), where *v*=±1 is the skyrmion vorticity describing the sense of rotation of the in-plane components of spins along a contour encircling the skyrmion centre^1^. The sum of the phases *φ*_1_+*φ*_2_+*φ*_3_ is either 0 or *π* and the *z*-component of spin in the centre of each skyrmion is *S*^*z*^=cos(*φ*_1_+*φ*_2_+*φ*_3_). The topological charge, *Q*, of skyrmions forming the crystal^1^,^22^ is given by *Q*=*v* cos(*φ*_1_+*φ*_2_+*φ*_3_)=±1. The spin direction in the skyrmion centre is opposite to that of the applied magnetic field. Because of the arbitrary sign of vorticity, the topological charge of skyrmions can be both +1 and −1 for any field direction^21^. This degree of freedom does not exist for skyrmions in chiral magnets and magnetic bubbles, for which *v*=+1 and *Q* =− sign(*h*). The SkX occupies a significant part of the phase diagram and all its boundaries are lines of first-order transitions.

### Instabilities of spiral states

To clarify the origin of multi-*q* states, we discuss a continuum model of the frustrated triangular magnet, applicable when the modulation period is much larger than the lattice constant (*q*<<1):


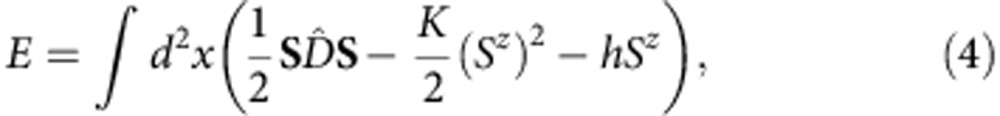


where the first term is the exchange energy, the second term is magnetic anisotropy and the last term is the Zeeman energy. The operator 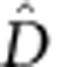 in the momentum representation can be expanded in powers of the wave vector **q**:


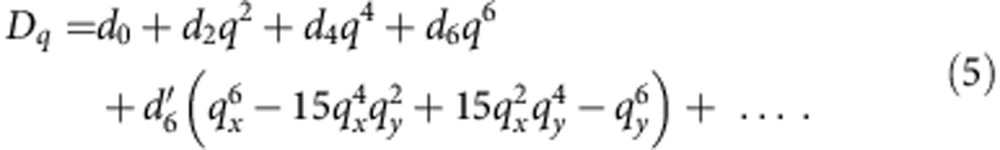


The anisotropy in the reciprocal space first appears in the sixth order of the expansion, resulting in 6 minima of *D*_**q**_ at **q**=±**q**_1_,±**q**_2_,±**q**_3_. It is convenient to add a constant to energy to make *D*_**q**_1__=0.

The sixfold degeneracy of the exchange energy minimum is the source of complexity. Another important factor is the relative rigidity of single-spiral states, which for *K*,*h*≠0 makes the states with several coexisting modulations, spiral or sinusoidal, energetically more favourable, even though the multi-*q* states have a larger exchange energy due to higher harmonics induced by the local constraint **S**^2^(**x**) = 1.

To prove this point, we consider the case of weak anisotropy, |*K*<<1|, and magnetic fields just below h_*_(*K*)=*D*_0_–*K*, at which the transition to the saturated FM state occurs. Then the in-plane component, **m**, of spin 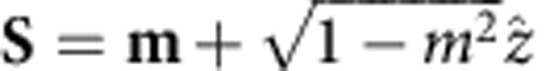 is small and the energy (4) can be expanded in powers of **m**:


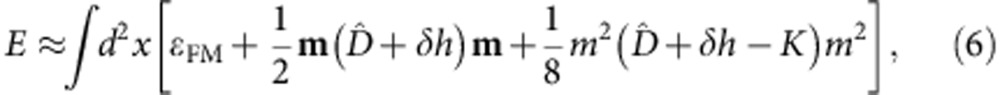


where *ɛ*_FM_ is the energy density of the saturated FM state and *δh*=h–h_*_(*K*)<0. For *K*<0, the ground state of the model is the CS: 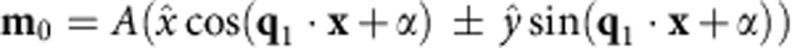 with 
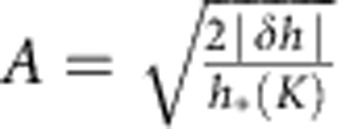
 and an arbitrary phase *α*. For *K*>0, the CS state becomes unstable against formation of an additional spin modulation. A small deviation from the CS, **k**=**m**-**m**_0_, changes energy by 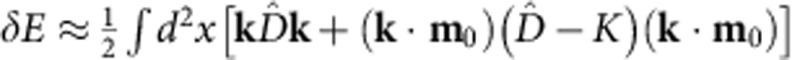. An additional modulation satisfying


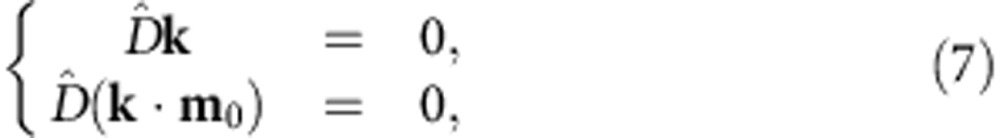


changes energy by 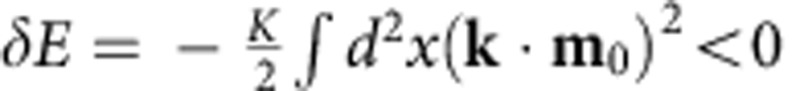, for *K*>0. The solution of equation (7) is the second *xy*-spiral with the wave vector **q**_2_ (or **q**_3_) and the opposite direction of spin rotation: 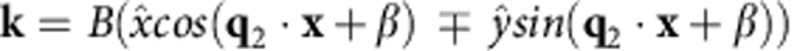. The two-spiral state has a lower energy because of the small sinusoidal modulation of the *z*-component of spin with the wave vector **q**_3_, *δS*^*z*^=−(**k**·**m**_0_)=−*AB* cos(**q**_3_·**x**−*α*−*β*), necessary to preserve the spin length. This sinusoidal modulation also has the minimal exchange energy, but its anisotropy energy for *K*>0 is lower than that of the *xy*-spirals, which makes the conical state unstable.

Next we discuss the instability of the VS state,


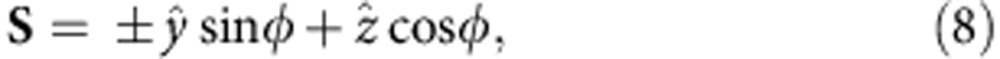


for *h*, *K*<<1. The magnetic field and easy axis parallel to the spiral plane deform the spiral: *φ*≈**q**_1_·**x**−*a* sin(**q**_1_·**x**)−*b* sin(2**q**_1_·**x**) with 
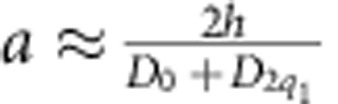
 and 
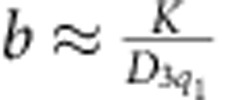
, corresponding to


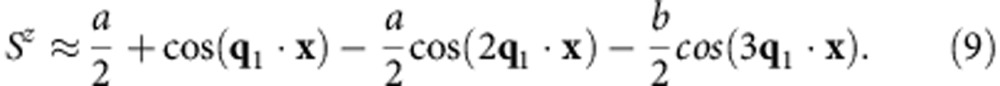


Importantly, the uniform magnetization equals to the amplitude of the energetically costly second harmonic. This makes the magnetic susceptibility of the *yz*-spiral low and leads to appearance of an *x*-component of spin above a critical magnetic field. Expanding the spin energy in powers of *S*^*x*^ around equation (8), we obtain 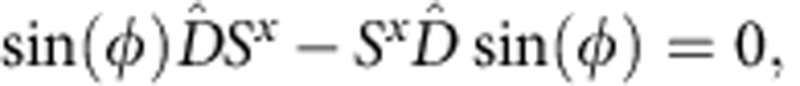 the solution of which is the sum of two sinusoidal spin modulations with the wave vectors **q**_2_ and **q**_3_ plus relatively small higher harmonics: 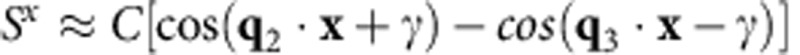, *γ* being an arbitrary phase. The second-order transition to the non-coplanar 3*q*-state (M-state) occurs at 
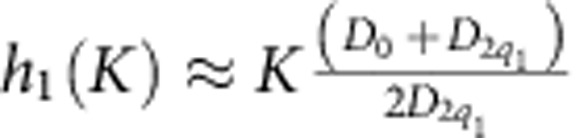
, when the lowering of the Zeeman energy due to the larger magnetization of the 3*q*-state compensates the increase of the anisotropy energy.

Similarly, the SkX owns its stability partly to the low anisotropy energy (as it is composed of three VSs) and partly to its large magnetic moment. The latter is the consequence of the constraint **S**^2^=1, which is impossible to maintain without adding to the 3*q*-state a large uniform magnetization. The same argument explains the enhanced stability of SkX to thin films of cubic chiral magnets^23^,^24^, where the term −*K*/2(*S*^*z*^)^2^ describes an effective surface anisotropy^17^. The anisotropy lowers the SkX energy with respect to the CS, while the applied magnetic field makes the SkX energetically more favourable than the helical spiral state.

An interesting question is why the 2*q* and skyrmion crystal states are favoured by both the entropic mechanism in absence of anisotropy^21^ and the magnetic anisotropy at zero temperature? The common origin seems to be the soft magnetic excitation spectrum of multi-*q* states, which on the one hand lowers free energy of these states by increasing their entropy and, on the other hand, lowers their energy by enhancing susceptibility to magnetic field and anisotropy.

The discussion of instabilities of spiral states in the continuum model of the frustrated triangular magnet also shows that the phase diagram Fig. 1 is generic, a minor difference being that in the discrete model with *J*_2_/*J*_1_=1/2, the wave vectors of the three fundamental modulations are commensurate with the lattice and constant throughout the phase diagram, while in the continuum model *q* is a function of *K* and *h*.

### Helicity reversals and interaction between skyrmions

Next we discuss properties of isolated skyrmions. The asymptotic of the in-plane spin components at a large distance *r* from the skyrmion centre is given by


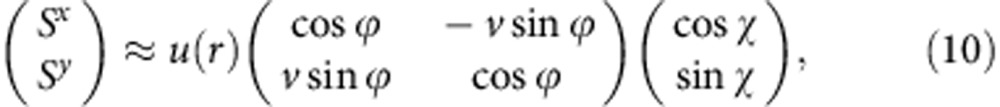


where *ϕ* is the azimuthal angle in the *xy*-plane and *v* and *χ* are, respectively, the skyrmion vorticity and helicity angle introduced in equation (3) (for simplicity, we consider large skyrmions and replace the discrete rotational symmetry of the triangular lattice by the continuous one).

In absence of in-plane magnetic anisotropy, the helicity angle *χ* is arbitrary, *χ*=0,*π* corresponding to skyrmions carrying a monopole moment, *A*∝∑_*j*_**x**_*j*_ ˙ **S**_*j*_, and *χ*=±*π*/2 corresponding to skyrmions with a toroidal moment, *T*^*z*^∝∑_*j*_[**x**_*j*_ × **S**_*j*_]^*z*^ (ref. 25). Arbitrary *χ* is a zero mode, which skyrmions in chiral magnets and magnetic bubbles do not have.

**Figure 3:**
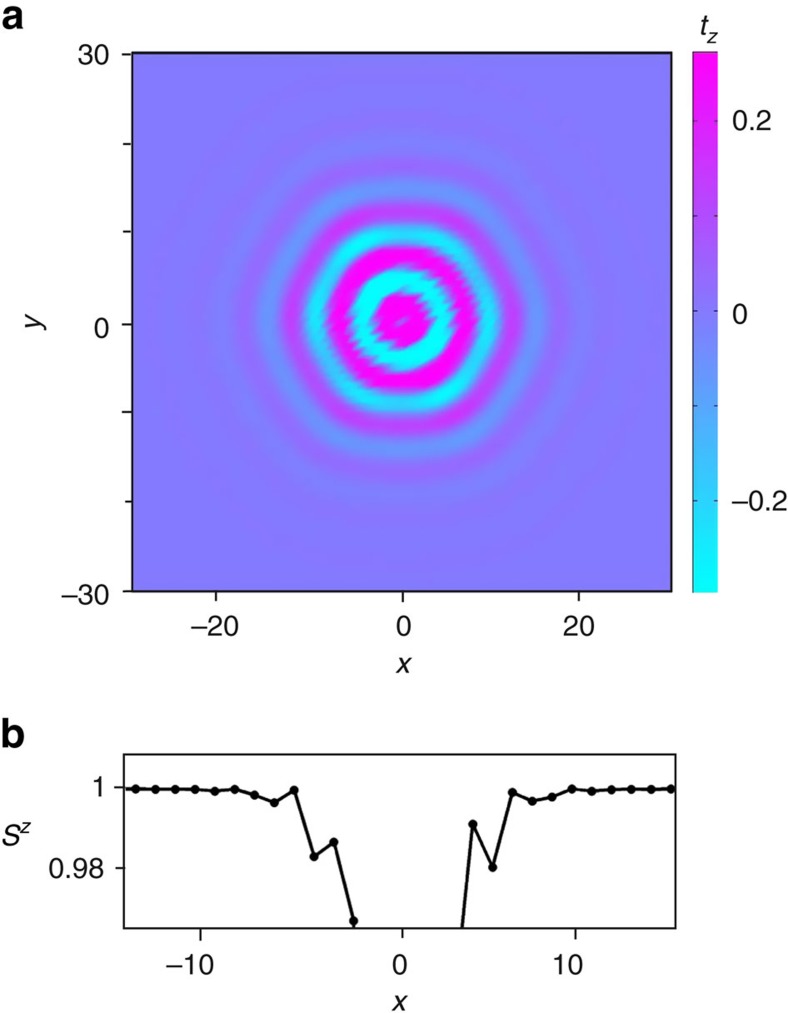
Helicity reversals. (**a**) False colour plot of the toroidal moment density, *t*_*z*_, in the skyrmion with the helicity angle *χ*=*π*/2. (**b**) Fan-like oscillations of the out-of-plane spin component, *S*^*z*^. The skyrmion centre is located at *x*=0.

In addition, the toroidal and monopole moment densities show periodic sign reversals as the distance *r* from the center increases (Fig. 3), similar to the helicity reversals observed in magnetic bubbles in a ferrimagnetic hexaferrite, where *χ* can be both +*π*/2 and −*π*/2 (ref. 26). We find it more convenient to keep the helicity angle fixed and associate these oscillations with the sign changes of *u*(*r*) in equation (10),


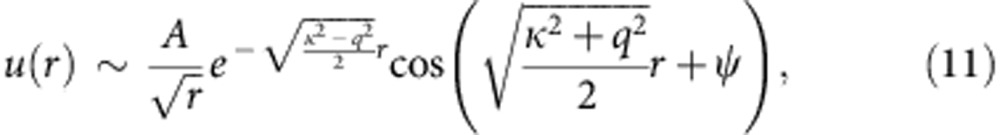


where 
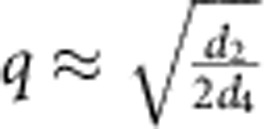
 is the length of the minimal-energy wave vector and 
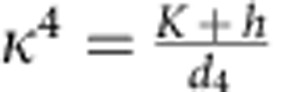
. The sign reversals of *u*(*r*) correspond to fan-like oscillations of spins around the *z* axis, whose amplitude decreases exponentially with *r* for *κ*>*q* or, equivalently, *h*>h_*_(*K*). This condition ensures that the skyrmion energy is finite, and, hence, the lower critical field for stability of isolated skyrmions coincides with the line separating the fully polarized state from the CS state, for *K*<0, and the 2*q′*-state, for *K*>0. There is also an upper critical field (dashed line in Fig. 1), above which skyrmions collapse.

**Figure 4:**
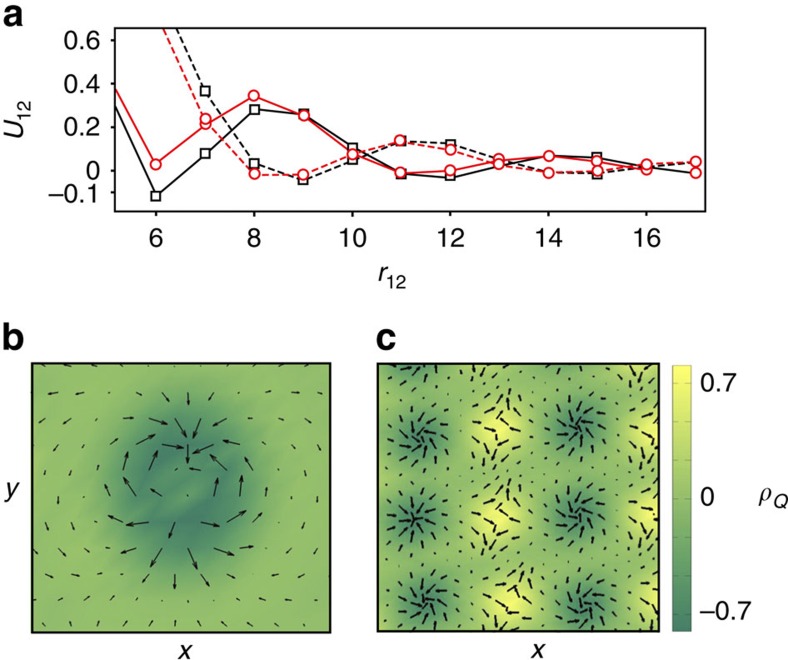
Unusual features of skyrmions in frustrated magnets. (**a**) The interaction energy, *U*_12_, versus the distance *r*_12_ between two skyrmions (black lines with rectangular markers), and skyrmion and antiskyrmion (red lines with circular markers). Solid (dashed) lines show the interaction for equal (opposite) helicities of the topological defects. (**b**) Metastable skyrmion with the topological charge *Q*=2. (**c**) Metastable skyrmion–antiskyrmion crystal with a rectangular lattice. In **b** and **c**, colour indicates the topological charge density, *ρ*_*Q*_, while arrows show the in-plane components of spins.

The fan oscillations of spins in skyrmion give rise to sign changes of the skyrmion–skyrmion interaction *U*(*r*_12_) (Fig. 4a). This interaction depends on the topological charges of skyrmions. For brevity, we call a skyrmion with positive vorticity and *Q*=−1 a skyrmion, while a skyrmion with *v*=−1 and *Q*=+1 is called an antiskyrmion. Two skyrmions or two antiskyrmions attract each other at distances of the order of the skyrmion diameter, the maximal reduction of energy being ∼10% of the skyrmion energy. Because of the attraction, the skyrmion crystal phase extends to *h*>h_*_(*K*), as the energy per skyrmion in the crystal can be negative even when the energy of an isolated skyrmion is positive. Isolated skyrmions tend to form clusters with a short-range crystal order, resembling the clustering of vortices in 1.5-type superconductors^27^. The oscillating interactions between skyrmions in the frustrated magnet are in sharp contrast with skyrmion–skyrmion interactions in non-centrosymmetric magnets, which do not show fan-like oscillations and repel each other at all distances^18^.

Interactions between skyrmions also depend on their helicities: *U*_12_(*r*)≈*V*(*r*)+*W*(*r*)cos(*χ*_1_−*χ*_2_) (Fig. 4a), which couples the helicity dynamics to the translational motion of skyrmions. Another difference from chiral magnets is the existence of the locally stable skyrmion with topological charge *Q*=±2, shown in Fig. 4b, which can be considered as a tightly bound state of two skyrmions with opposite helicities, similar to the magnetic bubble with *Q*=2 observed in thin films of a colossal magnetoresistance (CMR) manganite^28^. The energy of the *Q*=2 skyrmion is, however, larger than the energy of two *Q*=1 skyrmions.

The helicity dependence of the skyrmion–antiskyrmion interaction is more complex: it is approximately proportional to cos(2*α*_sa_+*χ*_s_−*χ*_a_), where *α*_sa_ is the angle between the line connecting skyrmion with antiskyrmion and the *x* axis, with respect to which the skyrmion and anstiskyrmion helicity angles, *χ*_s_ and *χ*_a_, are defined. Figure 4a shows skyrmion–antiskyrmion interactions for *χ*_s_=*χ*_a_ and *α*_sa_=0. The minimum of the skyrmion–antiskyrmion potential occurs at larger distances and is more shallow than that of the skyrmion–skyrmion potential. One can form a metastable rectangular crystal with alternating columns of skyrmions and antiskyrmions (Fig. 4c). Such a crystal with zero topological charge has a higher energy than a purely skyrmion (or purely antiskyrmion) crystal, which is important for observation of Topological Hall Effects in frustrated magnets.

### Magnetoelectric coupling

All modulated states in the phase diagram Fig. 1 break inversion symmetry and can induce an electric polarization, **P**, in a magnetic insulator. The two possible directions of spin rotation in spirals correspond to two opposite directions of **P** (ref. 29).

Consider, for example, a quasi-two-dimensional magnet with the triangular spin layers stacked directly on top of each other (the so-called eclipsed stacking^30^) and the symmetries of the crystal lattice being inversion *I*, threefold axis 3_*z*_ and the twofold axis 2_*x*_ (e.g. space groups 
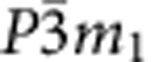
, 
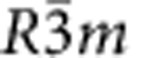
). This allows for three independent Lifshitz invariants describing the magnetically induced electric polarization, which in momentum representation have the form:


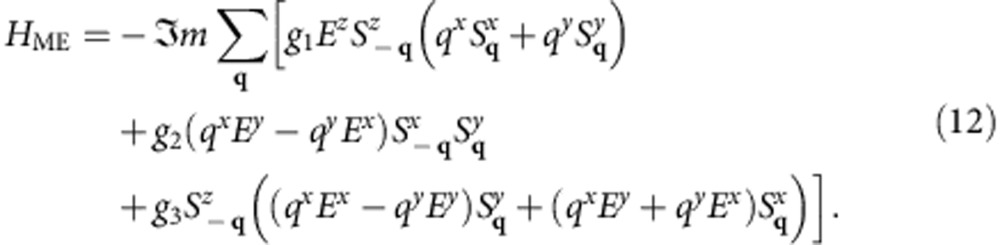


The polarization, 
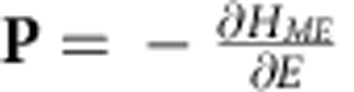
, described by the first two terms lies in the spiral plane and is orthogonal to the spiral wave vector, as is the case for **P** induced by a cycloidal spiral through the inverse Dzyaloshinskii–Moriya mechanism^31^,^32^,^33^. The third term describes other symmetry-allowed directions of polarization, in particular, **P** parallel to the wave vector of a helical spiral^34^. The magnititude of electric polarization induced by spiral magnetic orders varies widely: 2000 μĊm^−2^ in DyMnO_3_ (ref. 35) and 2 μĊm^−2^ in CoCr_2_O_4_ (ref. 36).

**Figure 5:**
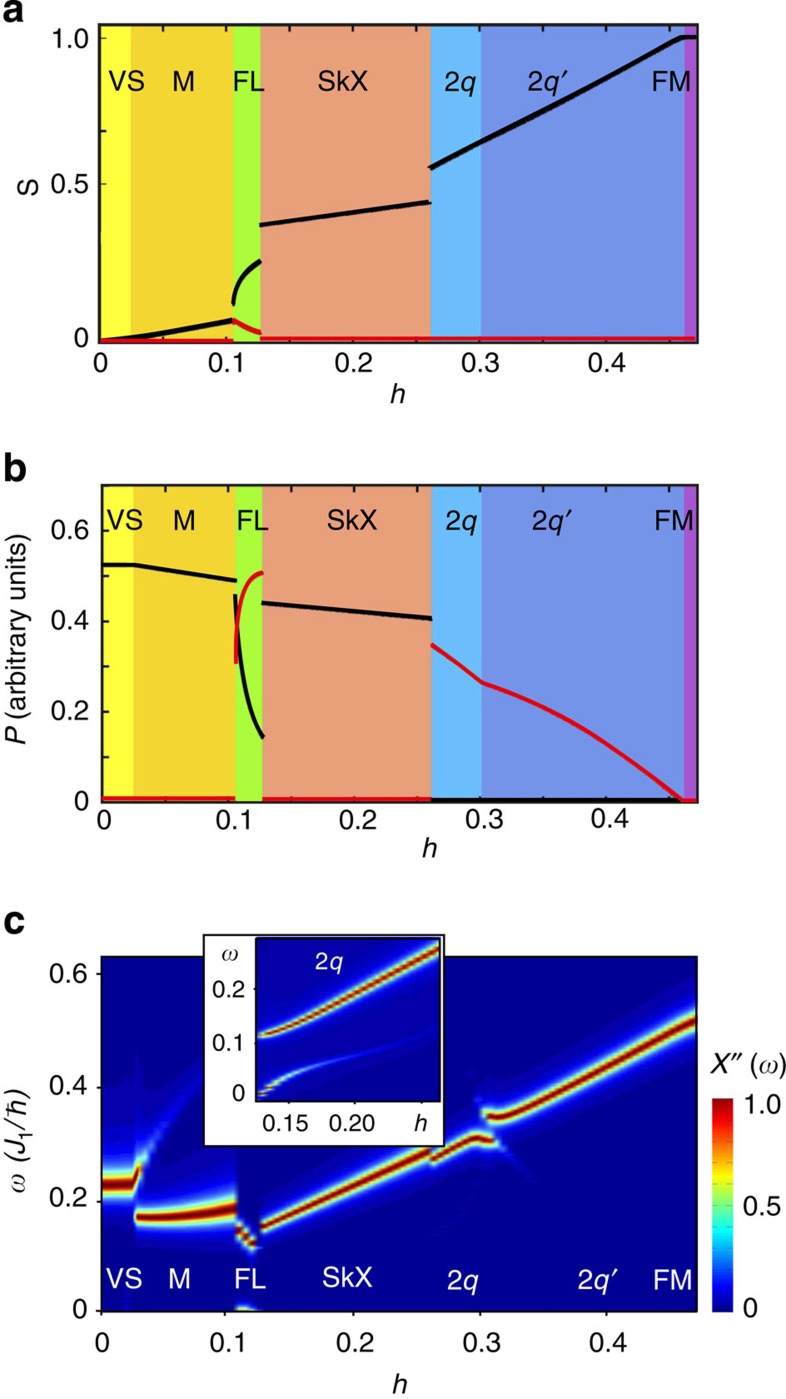
Multiferroic and dynamical properties of multi-*q* states. Magnetic field dependence at *K*=0.04 of (**a**) the average spin vector 〈**S**〉, (**b**) the electric polarization vector **P** and (**c**) the imaginary part of the in-plane magnetic susceptibility, *χ′′*(*ω*) (arbitrary units). Black and red lines in **a** and **b** are the out-of-plane and in-plane components of the vectors. The electric polarization was calculated using equation (12) for *g*_1_=*g*_2_ and *g*_3_=0. The inset in **c** shows magnetic modes in the 2*q*-state near the transition to the flop state.

Figure 5b shows magnetic field dependence of the electric polarization in arbitrary units, for *K* = 0.04, *g*_2_ = *g*_1_ and *g*_3_ = 0. The states with spirals in a vertical plane, including the SkX, induce polarization in the *z*-direction (black line), while the states with spirals in the horizontal plane (CS and the two-spiral states) induce an in-plane polarization (red line). In the flop phase the spiral plane rotates in an applied magnetic field and so does **P**. The spontaneous in-plane magnetization, present in this phase (Fig. 5a) allows for effective control of the polarization direction by magnetic field and *vice versa*.

The orientations of spiral planes and skyrmion helicity in realistic materials will be affected by the sixth-order in-plane magnetic anisotropy, which we neglected. The interplay between this weak anisotropy and the interaction of the electric polarization induced by the spiral with an applied electric field can lead to giant magnetoelectric effects, such as the electrically induced rotation of the skyrmion helicity.

### Magnetic resonance

We also studied collective spin dynamics for the states in the phase diagram Fig. 1 induced by the a.c. magnetic field, *h*_*ω*_sin(*ωt*), parallel to the *xy*-plane. Figure 5c shows the false colour plot of the imaginary part of the in-plane dynamical magnetic susceptibility calculated along the *K*=0.04 line in the phase diagram (as in panels (a) and (b)) by solving Landau–Lifshitz–Gilbert (LLG) equation (see Methods section). The phase transitions between different states are reflected in sudden changes in the magnetic resonance spectrum.

**Figure 6:**
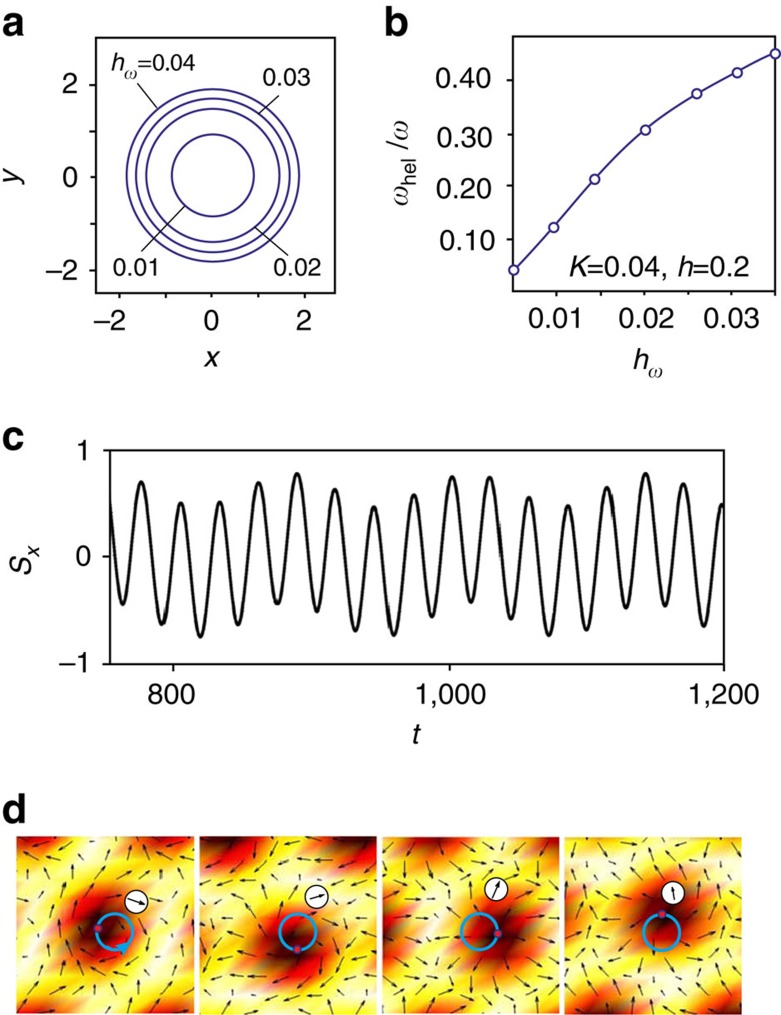
Coupled dynamics of skyrmion helicity and centre of mass. (**a**) Circular trajectories spanned by the centres of rotating skyrmions in the in-plane a.c. magnetic field for various amplitudes of the a.c. magnetic field *h*_*ω*_. (**b**) Frequency of the helicity rotation, *ω*_hel_, measured in units of the frequency of the a.c. field *ω*=0.224*J*_1_ℏ^−1^ versus *h*_*ω*_. (**c**) Time evolution of the *x*-component of spin at a chosen site, marked by a circle in the series of snapshots (**d**), which also shows current positions of the skyrmion centre (red point) and its trajectory for *h*_*ω*_=0.01. This calculation was performed for *h*=0.2 and *K*=0.04.

While the SkX in chiral magnets shows two resonances: one with the counterclockwise and another with the anti-counterclockwise rotation of the skyrmion centres^37^, in the frustrated magnet we find only one mode, in which skyrmions rotate counterclockwise. Figure 6a shows circular trajectories traced by the skyrmion centres for various amplitudes of the a.c. field and the resonant frequency *ω*=0.224*J*_1_ℏ^−1^.

The field-induced spin dynamics in frustrated magnets is, however, more complex than in chiral magnets, because of the additional zero mode—helicity. This can be seen from the fact that the time dependence of an in-plane component of spin at a chosen site (Fig. 6c) is a superposition of periodic oscillations with two different periods. The shorter period corresponds to the rotation of the skyrmion centre, whereas the longer one results from a monotonous increase of the helicity angle, as evidenced by the series of snapshots shown in Fig. 6d (Supplementary Movie 1). The helicity dynamics is induced by the rotation of skyrmion centres through a nonlinear coupling between the two modes, as can be seen from the nonlinear dependence of the frequency of helicity rotations, *ω*_hel_, on the amplitude of the a.c. magnetic field (Fig. 6b). The coupling between the helicity and translational dynamics must have important consequences for the current-induced motion of skyrmions in confined nanostructures^38^.

Another remarkable feature of the magnetic resonance spectrum is the presence of the zero-frequency peak in the flop phase (Fig. 5c) related to the non-zero spontaneous in-plane magnetization, which in absence of the in-plane magnetic anisotropy has an arbitrary direction. Surprisingly, a low-energy mode is also present in the 2*q*-state near the first-order transition to the flop phase, as shown in the inset to Fig. 5c (this transition does not actually occur at *K*=0.04 because of the intervening SkX phase, which was artificially removed to obtain the susceptibility shown in the inset).

## Discussion

In conclusion, the simultaneous presence of competing exchange interactions, uniaxial magnetic anisotropy and an applied magnetic field results in a plethora of multi-*q* states, including the skyrmion crystal. These complex magnetic states induce electric polarization. Soft magnetic modes present in some of these states can be used to control them with an electric field. Physics of isolated skyrmions in frustrated magnets is very rich and more complex than that in chiral magnets due to the additional degrees of freedom—vorticity and helicity. Skyrmion vorticity equal ±1 or ±2 can be used to store information. Skyrmion helicity interacts with an applied electric field and the coupling between the helicity and centre-of-mass dynamics of skyrmions leads to a characteristic magnetoelectric effect.

These results show that frustrated magnets can host skyrmions with physical properties not found in conventional non-centrosymmetric chiral magnets, provided they satisfy the following requirements: (1) spiral spin ordering in zero magnetic field resulting from competing exchange interactions; (2) threefold or sixfold symmetry axis, which ensures that the spirals with the three wave vectors, **q**_1_, **q**_2_ and **q**_3_ forming the skyrmion crystal, have the same energy; (3) **q**_1_+**q**_2_+**q**_3_=0; and (4) easy axis magnetic anisotropy axis stabilizing multi-*q* states.

Incommensurate magnetic orders resulting from the competition between NN FM and longer-range anti-FM interactions have been observed in triangular magnets with transition metal ions in edge-sharing octahedra, such as NiGa_2_S_4_ with strong third-NN interaction^39^,^40^, *α*-NaFeO_2_ with *J*_2_/*J*_1_∼1 (ref. 41) and magnetically induced electric polarization *P*=60 μĊm^−2^ (ref. 42) as well as in some dihalides, for example, Fe_*x*_Ni_1−*x*_Br_2_ (refs 43, 44, 45). Magnetocrystalline anisotropy of dihalides can be tuned by chemical substitutions: NiBr_2_ has an easy plane anisotropy *K*/*J*_1_=−0.1 (ref. 46), Fe_*x*_Ni_1-*x*_Br_2_ is an easy axis magnet for *x*>0.1 and FeBr_2_ is an Ising antiferromagnet with *K*/*J*_1_=1.3 (refs 45, 47). In *α*-NaFeO_2_ and dihalides, the wave vectors of three incommensurate modulations do not add to zero because they have an out-of-plane component. A combination of frustrated interactions in the basal plane with a FM coupling between spin layers would help to make **q**_1_, **q**_2_ and **q**_3_ coplanar.

## Methods

### Energy minimization

The energy (1) has been minimized using the iterative simulated annealing procedure and a single-step Monte-Carlo dynamics with the Metropolis algorithm (for details, see ref. 18). We imposed periodic boundary conditions and performed simulations for lattices of different sizes to check the stability of the numerical routine.

### Dynamical properties

The dynamical magnetic susceptibility for all multi-*q* states and the time evolution of spins in the oscillating magnetic field have been obtained by solving the LLG equation using the fourth-order Runge–Kutta method. We used a rather small dimensionless damping parameter, *α*=0.01, to make visible all peaks in the imaginary part of dynamical magnetic susceptibility in Fig. 5c. To obtain initial spin configurations, we used the annealing technique supplemented by a further LLG relaxation for *h*_*ω*_=0. When the convergence of the spin configurations was reached, we switched on a periodic magnetic field in the *xy*-plane to study the spin dynamics (Fig. 6) or applied a magnetic field pulse at *t*=0 and monitored the response to obtain the magnetic susceptibility (Fig. 5c).

### Interactions between topological defects

The skyrmion–skyrmion and skyrmion–antiskyrmion interaction potentials shown in Fig. 4 were calculated by minimizing the spin energy with the constraint, *S* =−1, imposed at the centres of the topological defects. To calculate the helicity dependence of the interactions, we constrained the helicity angle at six sites neighbouring to the skyrmion centre and imposed *S*^*z*^=−0.427 at these sites, which holds for an isolated skyrmion.

## Additional information

**How to cite this article:** Leonov, A. O. & Mostovoy, M. Multiply periodic states and isolated skyrmions in an anisotropic frustrated magnet. *Nat. Commun.* 6:8275 doi: 10.1038/ncomms9275 (2015).

## Supplementary Material

Supplementary Movie 1 Skyrmion helicity dynamics induced by ac magnetic field. Supplementary movie shows time evolution of the skyrmion crystal induced by the ac magnetic field, , parallel to the xy plane, obtained by solving numerically Landau-Lifshitz-Gilbert equation for , and . The clockwise rotation of skyrmion centres induces a relatively slow periodic variation of the skyrmion helicity.

## References

[b1] Nagaosa N. & Tokura Y. Topological properties and dynamics of magnetic skyrmions. Nat. Nanotechnol. 8, 899–911 (2013) . 24302027 10.1038/nnano.2013.243

[b2] Bogdanov N. & Yablonskii D. A. Thermodynamically stable “vortices”' in magnetically ordered crystals. The mixed state of magnets. Sov. Phys. JETP 68, 101–103 (1989) .

[b3] Mühlbauer S. . Skyrmion lattice in a chiral magnet. Science 323, 915–919 (2009) . 19213914 10.1126/science.1166767

[b4] Yu X. Z. . Real-space observation of a two-dimensional skyrmion crystal. Nature 465, 901–904 (2010) . 20559382 10.1038/nature09124

[b5] Dirac P. A. M. Quantized singularities in the electromagnetic field. Proc. R. Soc. A 133, 60 (1931) .

[b6] Skyrme T. H. R. A unified field theory of mesons and baryons. Nucl. Phys. 31, 556–569 (1962) .

[b7] Lee M., Kang W., Onose Y., Tokura Y. & Ong N. P. Unusual Hall anomaly in MnSi under pressure. Phys. Rev. Lett. 102, 186601 (2009) . 19518894 10.1103/PhysRevLett.102.186601

[b8] Neubauer A. . Topological Hall effect in the A phase of MnSi. Phys. Rev. Lett. 102, 186602 (2009) . 19518895 10.1103/PhysRevLett.102.186602

[b9] Zang J., Mostovoy M., Han J. H. & Nagaosa N. Dynamics of skyrmion crystals in metallic thin films. Phys. Rev. Lett. 107, 136804 (2011) . 22026888 10.1103/PhysRevLett.107.136804

[b10] Schulz T. . Emergent electrodynamics of skyrmions in a chiral magnet. Nat. Phys. 8, 301–304 (2012) .

[b11] Van Hoogdalem K. A., Tserkovnyak Y. & Loss D. Magnetic texture-induced thermal Hall effects. Phys. Rev. B 87, 024402 (2013) .

[b12] Mochizuki M. . Thermally driven ratchet motion of a skyrmion microcrystal and topological magnon Hall effect. Nat. Mater. 13, 241–246 (2014) . 24464244 10.1038/nmat3862

[b13] Fert A., Cros V. & Sampaio J. Skyrmions on the track. Nat. Nanotechnol. 8, 152–156 (2013) . 23459548 10.1038/nnano.2013.29

[b14] Iwasaki J., Mochizuki M. & Nagaosa N. Current-induced skyrmion dynamics in constricted geometries. Nat. Nanotechnol. 8, 742–747 (2013) . 24013132 10.1038/nnano.2013.176

[b15] Zhou Y. & Ezawa M. A reversible conversion between a skyrmion and a domain-wall pair in a junction geometry. Nat. Commun. 5, 4652 (2014) . 25115977 10.1038/ncomms5652

[b16] Tomasello E. M. R., Zivieri R., Torres L., Carpentieri M. & Finocchio G. A strategy for the design of skyrmion racetrack memories. Sci. Rep. 4, 6784 (2014) . 25351135 10.1038/srep06784 PMC4212245

[b17] Butenko A. B., Leonov A. A., Roessler U. K. & Bogdanov A. N. Stabilization of skyrmion textures by uniaxial distortions in noncentrosymmetric cubic helimagnets. Phys. Rev. B 82, 052403 (2010) .

[b18] Rößler U. K., Leonov A. A. & Bogdanov A. N. Chiral skyrmionic matter in non-centrosymmetric magnets. J. Phys. Conf. Ser. 303, 012105 (2011) .

[b19] Garel T. & Doniach S. Phase transitions with spontaneous modulation - the dipolar Ising ferromagnet. Phys. Rev. B 26, 325–329 (1982) .

[b20] Buhrandt S. & Fritz L. Skyrmion lattice phase in three-dimensional chiral magnets from Monte Carlo simulations. Phys. Rev. B 88, 195137 (2013) .

[b21] Okubo T., Chung S. & Kawamura H. Multiple-q states and the skyrmion lattice of the triangular-lattice Heisenberg antiferromagnet under magnetic fields. Phys. Rev. Lett. 108, 017206 (2012) . 22304286 10.1103/PhysRevLett.108.017206

[b22] Braun H.-B. Topological effects in nanomagnetism: from superparamagnetism to chiral quantum solitons. Adv. Phys. 61, 1–116 (2012) .

[b23] Yu X. Z. . Near room-temperature formation of a skyrmion crystal in thin-films of the helimagnet FeGe. Nat. Mater. 10, 106–109 (2011) . 21131963 10.1038/nmat2916

[b24] Huang S. X. & Chien C. L. Extended skyrmion phase in epitaxial FeGe(111) thin films. Phys. Rev. Lett. 108, 267201 (2012) . 23005010 10.1103/PhysRevLett.108.267201

[b25] Spaldin N. A., Fiebig M. & Mostovoy M. The toroidal moment in condensed-matter physics and its relation to the magnetoelectric effect. J. Phys. Condens. Matter 20, 434203 (2008) .

[b26] Yu X. Z. . Magnetic stripes and skyrmions with helicity reversals. Proc. Natl Acad. Sci. USA 109, 8856–8860 (2012) . 22615354 10.1073/pnas.1118496109 PMC3384203

[b27] Babaev E., Carlström A., Garaud B., Silaev A. & Speight J. M. Type-1.5 superconductivity in multiband systems: magnetic response, broken symmetries and microscopic theory. A brief overview. Physica C: Superconductivity 479, 2–14 (2012) .

[b28] Yu X. Z. . Biskyrmion states and their current-driven motion in a layered manganite. Nat. Commun. 5, 3198 (2014) . 24469318 10.1038/ncomms4198

[b29] Cheong S.-W. & Mostovoy M. Multiferroics: a magnetic twist for ferroelectricity. Nat. Mater. 6, 13–20 (2007) . 17199121 10.1038/nmat1804

[b30] Cava R. J. . in Introduction to Frustrated Magnetism 164, 131–154Springer Series in Solid-State Sciences (2011) .

[b31] Katsura H., Balatsky A. V. & Nagaosa N. Spin current and magnetoelectric effect in noncollinear magnets. Phys. Rev. Lett. 95, 057205 (2005) . 16090916 10.1103/PhysRevLett.95.057205

[b32] Sergienko I. A. & Dagotto E. Role of the Dzyaloshinskii-Moriya interaction in multiferroic perovskites. Phys. Rev. B 73, 094434 (2006) .

[b33] Mostovoy M. Ferroelectricity in spiral magnets. Phys. Rev. Lett. 96, 067601 (2006) . 16606047 10.1103/PhysRevLett.96.067601

[b34] Arima T. Ferroelectricity induced by proper-screw type magnetic order. J. Phys. Soc. Jpn 76, 073702 (2007) .

[b35] Goto T., Kimura T., Lawes G., Ramirez A. P. & Tokura Y. Ferroelectricity and giant magnetocapacitance in perovskite rare-earth manganites. Phys. Rev. Lett. 92, 257201 (2004) . 15245056 10.1103/PhysRevLett.92.257201

[b36] Yamasaki Y. . Magnetic reversal of the ferroelectric polarization in a multiferroic spinel oxide. Phys. Rev. Lett. 96, 207204 (2006) . 16803202 10.1103/PhysRevLett.96.207204

[b37] Mochizuki M. Spin-wave modes and their intense excitation effects in skyrmion crystals. Phys. Rev. Lett. 108, 017601 (2012) . 22304290 10.1103/PhysRevLett.108.017601

[b38] Zhou Y. . Dynamical magnetic skyrmions. *Preprint* at http://arxiv.org/abs/1404.3281 (2014) .

[b39] Nakatsuji S. . Spin disorder on a triangular lattice. Science 309, 1697–1700 (2005) . 16151004 10.1126/science.1114727

[b40] Stock C. . Neutron-scattering measurement of incommensurate short-range order in single crystals of the *S* =1 triangular antiferromagnet NiGa_2_S_4_. Phys. Rev. Lett. 105, 037402 (2010) . 20867806 10.1103/PhysRevLett.105.037402

[b41] McQueen T. . Magnetic structure and properties of the *S* =5/2 triangular antiferromagnet *α*-NaFeO_2_. Phys. Rev. B 76, 024420 (2007) .

[b42] Terada N. . Magnetic and ferroelectric orderings in multiferroic *α*-NaFeO_2_. Phys. Rev. B 89, 184421 (2014) .

[b43] Day P., Dinsdale A., Krausz E. R. & Robbins D. J. Optical and neutron diffraction study of the magnetic phase diagram of NiBr_2_. J. Phys. C 9, 2481–2490 (1976) .

[b44] Régnault L. P., Rossat-Mignod J., Adam A., Billerey D. & Terrier C. Inelastic neutron scattering investigation of the magnetic excitations in the helimagnetic state of NiBr_2_. J. Phys. 43, 1283–1290 (1982) .

[b45] Moore M. W. & Day P. Magnetic phase diagrams and helical magnetic phases in M_*x*_Ni_1−*x*_Br_2_ (M=Fe, Mn): A neutron diffraction and magneto-optical study. J. Solid State Chem. 59, 23–41 (1985) .

[b46] Tuchendler J. & Katsumata K. Helimagnetic resonance experiments in NiBr_2_ at millimetre wavelength. Solid State Commun. 8, 769–770 (1985) .

[b47] Yelon W. B. & Vettier C. Neutron scattering study of the magnetic excitations in FeBr_2_. J. Phys. C 8, 2760 (1975) .

